# Association of Fluid Management With Mortality of Sepsis Patients With Congestive Heart Failure: A Retrospective Cohort Study

**DOI:** 10.3389/fmed.2022.714384

**Published:** 2022-03-02

**Authors:** Ning Dong, Nan Gao, Wenxin Hu, Yuhang Mu, Li Pang

**Affiliations:** ^1^Department of Emergency, The First Hospital of Jilin University, Changchun, China; ^2^Department of Emergency, The Third Affiliated Hospital of Changchun University of Chinese Medicine, Changchun, China; ^3^The Affiliated Hospital of Changchun University of Chinese Medicine, Changchun, China

**Keywords:** sepsis, fluid accumulation, fluid balance, heart failure, mortality

## Abstract

Sepsis management includes intravenous fluid (IVF) resuscitation, but patients with pre-existing congestive heart failure (CHF) have a higher risk of fluid overload. Further, patients with sepsis with concomitant CHF present worse clinical outcomes. Nevertheless, there is limited evidence of the association between fluid management and the outcomes of patients with concomitant sepsis and CHF. This retrospective cohort study aimed to evaluate the association between fluid management and in-hospital mortality in patients with sepsis and concomitant heart failure (HF). The patients' data were extracted from the Multi-parameter Intelligent Monitoring in Intensive Care III Database. The primary outcome was in-hospital mortality. A restricted cubic spline model was used to explore the relationship between variables and in-hospital mortality. Logistic models were built using the linear spline function and design variables to investigate the association of fluid balance (FB), fluid intake (FI), and fluid accumulation index (FAI, calculated as the FB/FI ratio) with mortality. Overall, 1,801 patients were included. The overall mortality rate was 27.7%. After adjusting for confounding variables, FAI was found to be associated with in-hospital mortality, whereas FB and FI were not. With FAI values of 0–0.42 set as references, FAI values <0 were not associated with in-hospital mortality [odds ratio (OR): 1.078; 95% confidence interval (CI): 0.774–1.503], whereas FAI values > 0.42 were significantly associated with higher in-hospital mortality (OR: 1.461; 95% CI: 1.099–1.954). High FAI values (>0.42) were associated with high in-hospital mortality in patients with sepsis with HF, while FB and FI were not. Proper fluid management may improve the outcomes of patients with sepsis and concomitant HF.

## Introduction

Sepsis is a complex clinical disorder with a high risk of death due to acute organ dysfunction arising from dysregulated host response to an infection (1). The guidelines for sepsis management recommend early administration of antibiotics, source control, and intravenous fluid (IVF) resuscitation (2). However, a sustained positive fluid balance (FB) during intensive care unit (ICU) stay is also associated with higher mortality rates in patients with sepsis (3–6). In addition, patients with congestive heart failure (CHF) may be more sensitive to IVF administration and therefore, are at risk of fluid overload (7, 8). However, current evidence on the influence of fluid resuscitation on the outcomes of patients with sepsis with pre-existing heart failure (HF) is mainly focused on the outcomes of sepsis bundle implementation. The method to evaluate the status of fluid overload and how fluid accumulation is associated with outcomes remains unknown in these patients.

This retrospective cohort study aimed to explore the association between fluid management and in-hospital mortality in patients with sepsis and concomitant HF, as well as to find a better indicator to guide fluid management among FB, fluid intake (FI), and the fluid intake ratio (FB/FI), which is called the fluid accumulation index (FAI) and was first introduced by Shen et al. (9). We focused on fluid management within the first 48 h after admission to the ICU and explored the relationship of FI, FB, and FAI with in-hospital mortality in sepsis patients with pre-existing HF.

## Materials and Methods

### Study Design and Database

The participants were patients identified from the Medical Information Mart for Intensive Care III (MIMIC-III) database (10, 11), which is a large, freely-available database containing de-identified health-related data of >40,000 patients who were admitted to the critical care units of the Beth Israel Deaconess Medical Center between 2001 and 2012. The database includes information concerning demographic characteristics, bedside vital signs (approximately one data point per hour), laboratory test results, procedures, medications, caregiver notes, imaging reports, and mortality (both in and out of the hospital).

The database is accessible to researchers who have completed a protecting human subjects training. The institutional review boards of the Massachusetts Institute of Technology (Cambridge, MA) and Beth Israel Deaconess Medical Center (Boston, MA) approved the establishment of the database. Thus, consent was obtained for the original data collection but not specifically for this study. Ning Dong, the first author of this study, extracted the data, as he had completed the online training course by the National Institutes of Health (certification number: 9135690).

### Patient Selection

The inclusion criteria were as follows: 1) age >18 years; 2) ICU admission for >48 h; and 3) meeting the sepsis diagnostic criteria stipulated by the recommendation in the Surviving Sepsis Campaign 2016 (12). This was defined as life-threatening organ dysfunction [total Sequential Organ Failure Assessment (SOFA) score ≥2 points], caused by a dysregulated host response infection. Accordingly, patients were considered to have sepsis if they were suspected of having infection at admission and had a SOFA score ≥2 points (evaluated within 24 h after admission). Suspected infection, which was defined as the acquisition of a body fluid culture temporally contiguous to the administration of antibiotics shortly after ICU admission (13), was used to refer to cases with an identified infection. CHF was identified using the International Classification of Diseases code 9, following the study by Quan et al. (14) (Supplementary Table 1).

The exclusion criteria were as follows: 1) no fluid/output records within the first 48 h of admission, 2) initiation of renal replacement therapy within 48 h after ICU admission, and 3) cardiac surgery under cardiopulmonary bypass.

For patients who were admitted to the ICU more than once, only data from the first ICU stay were analyzed. A schematic illustration of the study design is presented in Supplementary Figure 1.

### Data Extraction

Data were extracted using pgAdmin 4 version 4.26 (Copyright 2013–2020, The pgAdmin Development Team). To improve the reproducibility of our study, we used the MIMIC Code Repository (15), which provides an open-source code alongside the freely accessible MIMIC-III database, to identify study cohorts and outcomes. This included calculation of the SOFA score, identification of suspected infection, classification of chronic comorbidities, and identification of in-hospital mortality. Other extracted data included demographic characteristics; vital signs (on day 1 after admission to the ICU); laboratory outcomes (on day 1 after admission to the ICU); vasopressor use (if any, after admission to the ICU); FI and fluid output; FB, calculated as fluid intake minus fluid output; and FAI, calculated as fluid balance divided by fluid intake (more information is presented in the Supplementary File concerning the Structured Query Language (SQL) codes used in the extraction of fluid management), as well as the types of ICU (e.g., coronary care unit, surgical ICU). Because of the retrospective design of the present study, the onset time of sepsis was not known; therefore, fluid management before admission was not included in the analysis.

### Statistical Analysis

Continuous variables are presented as means (standard deviations) or medians (interquartile ranges), as appropriate. Meanwhile, categorical variables are presented as proportions. Student's *t*-test, the Wilcoxon rank-sum test, or Pearson's chi-square test was used as appropriate. The primary endpoint was in-hospital mortality. The percentages of missing values are presented in Supplementary Table 2. Variables with missing rates >10% were removed from the final analysis. Lactic acid was excluded from the final analysis because >20% of the patients had missing data. Other missing values of variables were imputed using the R package “missForest” (R Foundation for Statistical Computing, Vienna, Austria) (16). Boxplots were used to detect the outliers of FI and fluid output. A total of 133 (133/1,934, 6.9%) cases with outliers were identified and removed from the logistic regression analysis. Univariate logistic regression was used to identify variables associated with in-hospital mortality. Further, a stepwise backward elimination method was used to remove variables with *p* > 0.2. We kept removing and adding variables according to their impact on the coefficient of the other variables until all variables that remained in the model were clinically and statistically significant, and the fit of these models was then tested using the partial likelihood ratio test (17). Multivariate logistic regression using the “backward” stepwise method was performed to adjust for confounding factors. Linear spline (LSP) and restricted cubic spline (RCS) functions were used to explore the relationship between in-hospital mortality and continuous confounding variables. The number of knots (knots = 3, 4, or 5) of RCS-transformed models was selected using the minimum Akaike information criterion (AIC) (18) (Supplementary Figure 2, Supplementary Table 3). Multi-collinearity was checked against the variance inflation factor (Supplementary Tables 4, 5). All statistical analyses were performed using R software version 4.0.3. R packages “missForest (16),” “rms (19),” and “Hmsic (20)” were used during analyses. A two-tailed *p* < 0.05 was considered statistically significant.

### Ethics Approval and Consent to Participate

The institutional review boards of the Massachusetts Institute of Technology (Cambridge, MA) and Beth Israel Deaconess Medical Center (Boston, MA) approved the establishment of the database. Thus, consent was obtained for the original data collection but not specifically for this study.

## Results

### Patient Characteristics

A total of 1,801 patients with sepsis with CHF, including 1,302 survivors and 499 non-survivors, were included in this analysis (Table 1). The in-hospital mortality rate was 27.7%. The comparison of baseline characteristics between survivors and non-survivors is presented in Table 1. Non-survivors showed significantly higher FB (3.2 L/48 h vs. 1.7 L/48 h, *p* < 0.001), FI (5.8 L/48 h vs. 5.2 L/48 h, *p* < 0.001), and FAI (0.55 vs. 0.34, *p* < 0.001) and a significantly lower urine output (2.2 L/48 h vs. 3 L/48 h, *p* < 0.001) than survivors.

**Table 1:** Comparison of patient characteristics between survivors and non-survivors.

	**Survivors** **(*n* = 1,302)**	**Non-survivors** **(*n* = 499)**	***p*-value**
**Demographics**			
Age, years	76.7 (64.3–83.9)	79 (68.7–85.6)	0.002
Male sex, *n* (%)	619 (47.5)	247 (49.5)	0.489
Weight, kg	77 (63.5–93)	74.9 (62–90)	0.052
**ICU type**, ***n*** **(%)**			
CCU	282 (21.7)	115 (23.0)	0.646
CSRU	58 (4.5)	22 (4.4)	
MICU	691 (53.1)	274 (54.9)	
SICU	166 (12.7)	52 (10.4)	
TSICU	105 (8.1)	36 (7.2)	
SOFA, 1st day	5 (3–7)	6 (4–9)	<0.001
SAPS II, 1st day	42 (34.25–50)	48 (41–57)	<0.001
**Chronic comorbidities**, ***n*** **(%)**			
Hypertension	750 (57.6)	259 (51.9)	0.033
Diabetes with complication	110 (8.4)	37 (7.4)	0.535
Valvular disease	249 (19.1)	88 (17.6)	0.511
COPD	444 (34.1)	176 (35.3)	0.68
Kidney disease	334 (25.7)	119 (23.8)	0.466
Liver disease	119 (9.1)	78 (15.6)	<0.001
**Laboratory indexes, 1st day**			
White blood cells, per 10^9^/L	13.2 (9.7–18.2)	14.2 (9.7–19.3)	0.264
Hemoglobin, g/dL	9.9 (8.7–11.2)	9.7 (8.6–11)	0.026
Platelets, per 10^9^/L	199 (143–269)	180 (118–254)	<0.001
Serum creatinine, mg/dL	1.3 (0.9–2)	1.50 (1.1–2.2)	<0.001
Blood urea nitrogen, mg/dL	31 (20–47)	39 (24.3–59.5)	<0.001
Creatinine clearance rate, mL/min	46(28.3–74.7)	37.8(24.6–60.2)	<0.001
Sodium, mmol/L	137 (134–141)	137 (134–140)	0.093
Potassium, mmol/L	3.80 (3.4–4.2)	3.8 (3.5–4.3)	0.012
Chlorine, mmol/L	107 (103–110)	106 (102–111)	0.249
International normalized ratio	1.4 (1.2–1.8)	1.4 (1.2–2)	0.012
Lactic acid, mmol/L	2 (1.4–3.2)	2.3 (1.6–3.70)	<0.001
**Vital sign, 1st day**			
Mean heart rates, per min	85.5 (74.3–98.5)	88.3 (75.3–100)	0.089
Minimum mean arterial pressure, mmHg	55 (48–62)	53 (46–60)	0.002
**Hemodynamic indexes**			
Fluid intake, L/48h	5.2 (3.3–7.9)	5.8 (3.8–8.5)	0.001
Urine output, L/48h	3 (2–4.4)	2.2 (1.4–3.5)	<0.001
Fluid balance, L/48h	1.7 (−0.5–4.7)	3.2 (4.5–6)	<0.001
Fluid accumulation index, 48h	0.34 (−0.15–0.65)	0.55 (0.14–0.78)	<0.001
**Vasoactive agents**, ***n*** **(%)**			
Dopamine	152 (11.7)	91 (18.2)	<0.001
Dobutamine	45 (3.5)	31 (6.2)	0.013
Norepinephrine	316 (24.3)	179 (35.9)	<0.001
Epinephrine	14 (1.1)	3 (0.6)	0.51
Length of ICU, days	5.1 (3.1–10.8)	7.5 (4.5–13.2)	<0.001

*CCU, coronary care unit; CSRU, cardiac surgery recovery unit; MICU, medical intensive care unit; SICU, surgical intensive care unit; TSICU, trauma/surgical intensive care unit; SOFA, Sequential Organ Failure Assessment; SAPS II, Simplified Acute Physiology Score II; COPD, chronic obstructive pulmonary disease; ICU, intensive care unit*.

### Univariable Logistic Regression Analysis of the Association Among FI, FB, FAI, and In-hospital Mortality

**Figure 1:**
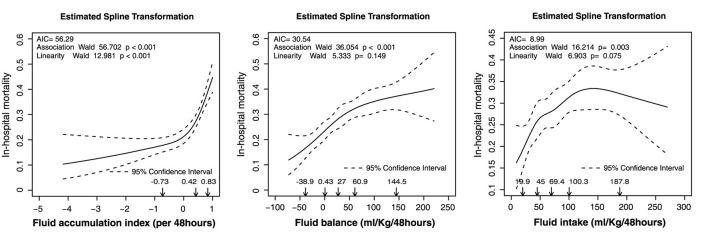
Estimated spline transformation of FI, FB, and FAI for in-hospital mortality. AIC, Akaike information criterion; FAI, fluid accumulation index; FB, fluid balance; FI, fluid intake.

The relationship of FI, FB, and FAI with in-hospital mortality is presented in Figure 1. The number of knots of the RCS function of FAI, FI, and FB, which had a minimum AIC, was 3, 5, and 5, respectively (Supplementary Table 3). For better interpretation of the coefficients in the regression model, we used the LSP function and the designed variables in the univariable logistic regression. The knot selection of the LSP function and the designed variables were according to the shape of the RCS regression curve, and the knots were detected using the RCS function (Table 2). The unadjusted odds ratios (ORs) of FI, FB, and FAI are presented in Table 2. Compared with moderate FI (45–100 mL/kg/48 h), low FI (<45 mL/kg/48 h) was significantly associated with in-hospital mortality [OR: 0.682, 95% confidence interval (CI): 0.523–0.887]. However, there was no significant association between FI and in-hospital mortality according to the LSP function. With moderate FB (0–60 mL/kg/48 h) as reference, low FB (≤ 0 mL/kg/48 h) was significantly associated with a lower in-hospital mortality (OR: 0.694; 95% CI: 0.529–0.905), while high FB (>60 mL/kg/48 h) was associated with a higher in-hospital mortality (OR: 1.427; 95% CI: 1.123–1.813). When using the LSP function, FB ≤ 0 mL/kg/48 h (OR: 1.012; 95% CI 1.002–1.023) and 0–60 mL/kg/48 h (OR: 1.007; 95% CI: 1.002–1.013) were significantly associated with a high in-hospital mortality. With FAI 0–0.42 as reference, FAI >0.42 was significantly associated with a high in-hospital mortality (OR: 1.725; 95% CI: 1.322–2.266). When using the LSP function, FAI ≤ 0 (OR: 1.454; 95% CI: 1.117–1.989) and FAI >0.42 (OR: 8.859; 95% CI: 4.002–19.775) were significantly associated with a high in-hospital mortality.

**Table 2:** Univariable logistic regression analysis for in-hospital mortality using linear spline function and the designed variables.

**Variables**	**Crude odds** **ratio**	**95% confidence** **interval**	***p*-value**
**Fluid intake (mL/kg/48 h)**			
**Using linear spline function**			
≤ 45	1.014	0.998–1.032	0.086
45–100	1.006	1–1.013	0.062
>100	1	0.996–1.003	0.825
**As designed variables**			
≤ 45	0.682	0.523–0.887	0.005
45–100 as reference			
>100	1.249	0.981–1.589	0.071
**Fluid balance (mL/kg/48 h)**			
**Using linear spline function**			
≤ 0	1.012	1.002–1.023	0.025
0–60	1.007	1.002–1.013	0.012
>60	1.002	0.998–1.006	0.237
**As designed variable**			
≤ 0	0.694	0.529–0.905	0.007
0–60 as reference			
>60	1.427	1.123–1.813	0.004
**Fluid accumulation index (per 48 h)**			
**Using linear spline function**			
≤ 0	1.454	1.117–1.989	0.011
0–0.42	0.724	0.294–1.774	0.48
>0.42	8.859	4.002–19.775	<0.001
**As designed variables**			
≤ 0	0.886	0.645–1.218	0.455
0–0.42 as reference			
>0.42	1.725	1.322–2.266	<0.001

### Association Between Other Confounders and In-hospital Mortality

The association between other confounders and in-hospital mortality was explored using RCS transformation. The linear Wald test showed that only platelet counts needed transformation (p < 0.001, Supplementary Table 3, Supplementary Figure 3). The ORs and 95% CIs of other confounding variables in the univariable logistic regression are presented in Supplementary Table 4.

### Association Among FI, FB, FAI, and In-hospital Mortality After Adjusting for Other Confounding Factors

**Figure 2:**
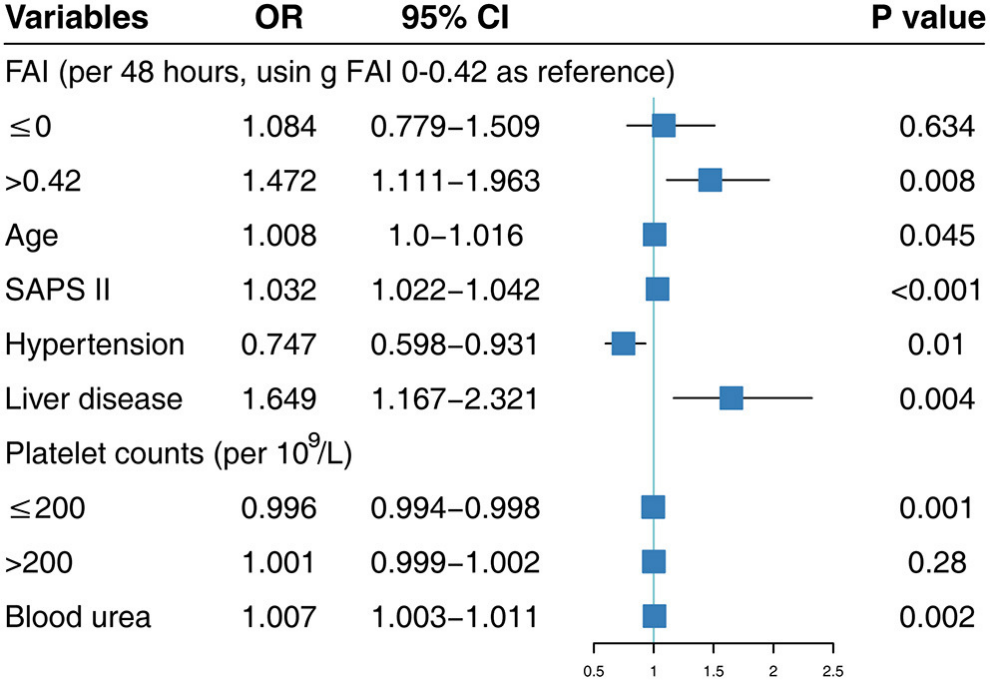
Multivariable logistic regression using designed variables. SAPS II, Simplified Acute Physiology Score II; FAI, fluid accumulation index; OR, odds ratio; CI, confidence interval.

We pooled FI, FB, FAI, and other confounders together into a stepwise multivariable logistic regression model using the LSP function and the designed variables (Supplementary Tables 5, 6). FAI, but not FB and FI, was significantly associated with in-hospital mortality in these two multivariable logistic regression models. After adjusting for the related confounders listed in Figure 2, with FAI 0–0.42 as reference, FAI values >0.42 were significantly associated with in-hospital mortality (OR: 1.472; 95% CI: 1.111–1.963). We found a similar association between FAI and in-hospital mortality in the LSP-transformed multivariable logistic regression analysis. Values of FAI ≤ 0 (OR: 1.408; 95% CI: 1.088–1.915) and FAI >0.42 (OR: 4.683; 95% CI: 2.018–10.927) were significantly associated with a higher in-hospital mortality (Supplementary Table 5). To further explore the association among FI, FB, and in-hospital mortality, we forced FI or FB and other confounders into stepwise multivariable regression and found that FB and FI were not significantly associated with in-hospital mortality after adjusting for other related confounders (Supplementary Tables 7, 8).

## Discussion

In this study, we retrospectively analyzed the association between fluid management (FI, FB and FAI) and in-hospital mortality in sepsis patients with HF. After adjusting for related confounders in multivariable logistic regression, we found that a high FAI (>0.42 per 48 h), but not FI and FB, was significantly associated with a high in-hospital mortality in patients with sepsis with HF.

To the best of our knowledge, this is the first study on the association between fluid accumulation and in-hospital mortality in a large cohort of patients with sepsis with HF. The core management of sepsis seems to be a paradox in patients with HF as the fluid bolus and vasoactive agents required in sepsis raise concerns in the context of cardiac dysfunction in such cases (21). These patients have worse clinical outcomes (22), even after hospital discharge (23). However, there is limited evidence on the influence of fluid resuscitation on the outcomes of patients with sepsis with pre-existing HF (21). Currently, no guideline recommendations are available for the management of patients with co-existing sepsis/septic shock and HF.

The 2016 Surviving Sepsis Campaign guideline issued a recommendation for using a minimum of 30 mL/kg (ideal body weight) of intravenous crystalloids in initial fluid resuscitation (2). Previous studies on fluid management in patients with sepsis with HF have mainly focused on this bundle implementation (24–26). Most sepsis patients require continued fluid administration following initial resuscitation. Such administration needs to be balanced with the risk of fluid accumulation and potential harm associated with fluid overload, including prolonged ventilation, progression of acute kidney injury, and increased mortality (27). However, the association between fluid accumulation and in-hospital mortality in patients with sepsis with HF remains unclear to date.

Fluid accumulation is associated with high mortality in patients with sepsis. Kelm et al. evaluated patients with severe sepsis and septic shock (with or without HF) treated with early goal-directed therapy and found that persistent fluid overload is common and associated with a higher use of medical interventions (thoracentesis and diuretics) and hospital mortality (28). Persistence of a positive daily FB over time is strongly associated with a high mortality rate in patients with sepsis (3). Early negative FB is independently associated with a better prognosis of patients with sepsis complicated with acute respiratory distress syndrome (29). FB and FI are usually analyzed separately when investigating the association between fluid management and outcomes in such patients. However, these two variables may be correlated to each other; thus, their separate analysis without adjustment might result in the overestimation of their significance. The definition of FAI was first introduced in the analysis of fluid management by Shen et al., who consequently found that the impact of FB on mortality is mediated by FAI in patients with sepsis (9). Our pooled FB, FI, and FAI analysis using multivariable logistic regression also showed that FAI, but not FB, was significantly associated with in-hospital mortality, after adjusting for confounding factors. This finding indicated that FAI may be a new clinical indicator for fluid management in patients with sepsis with CHF. With values of FAI ≤ 0 as reference, FAI values >0.42 were associated with a higher in-hospital mortality, whereas FAI values of 0–0.42 were not. Collectively, our results support that a meaningful level of fluid accumulation is associated with in-hospital mortality in patients with sepsis with HF. This finding provided useful preliminary evidence for fluid management in these patients.

Our study had some limitations. First, our retrospective study design may lead to a certain degree of information bias. For example, the value of lactic acid was removed from the final analysis due to the high rate of missing data. Second, this was a single-center study, and the applicability of the cutoff value of FAI remains unclear and needs to be validated in future prospectively designed studies. Third, fluid management before admission was not included in the data analysis, which could be a potential bias of this study. Fourth, the International Classification of Diseases codes, instead of the actual left ventricular ejection fraction, were used in identifying patients with HF. The association between compensatory or stage of heart function and patients' outcomes could not be evaluated in the study. Thus, the cutoff value of our study needs to be validated in future studies. Finally, MIMIC-III recorded data across 12 years (2001–2012), during which major changes have been made in sepsis guidelines (i.e., the fluid management might have been more liberal in those years). Thus, therapeutic bias should be considered when interpreting our findings. Subgroup analyses based on cardiac function may help us to further understand FAI and patients' outcomes. Further studies are required to investigate the underlying mechanisms and to validate our findings.

## Conclusion

A high FAI (FB/FI ratio) was found to be associated with high in-hospital mortality in patients with sepsis with HF. Thus, proper fluid management in these patients may improve outcomes.

## Data Availability Statement

The raw data supporting the conclusions of this article will be made available by the authors, without undue reservation.

## Ethics Statement

The database is accessible to researchers who have completed a protecting human subjects training. The institutional review boards of the Massachusetts Institute of Technology (Cambridge, MA) and Beth Israel Deaconess Medical Center (Boston, MA) approved the establishment of the database. Thus, consent was obtained for the original data collection but not specifically for this study.

## Author Contributions

ND performed data extraction, study design, and draft writing of the manuscript. NG, WH, and YM performed all statistical analyses and revised the manuscript for important intellectual content. LP performed data analysis and interpretation and revised the manuscript for the final version. All authors gave final approval of the version to be published and agreed to be accountable for all aspects of the work in ensuring that questions related to the accuracy or integrity of any part of the work are addressed. All authors contributed to the article and approved the submitted version.

## Conflict of Interest

The authors declare that the research was conducted in the absence of any commercial or financial relationships that could be construed as a potential conflict of interest.

## Publisher's Note

All claims expressed in this article are solely those of the authors and do not necessarily represent those of their affiliated organizations, or those of the publisher, the editors and the reviewers. Any product that may be evaluated in this article, or claim that may be made by its manufacturer, is not guaranteed or endorsed by the publisher.

## Abbreviations

AIC: Akaike information criterion
CHF: Congestive heart failure
CI: Confidence interval
FAI: Fluid accumulation index
FB: Fluid balance
FI: Fluid intake
ICU: Intensive care unit
IVF: Intravenous fluid
OR: Odds ratio
RCS: Restricted cubic spline
SOFA: Sequential organ failure assessment
SQL: Structured query language.
