# Supplementary material for: Association of Fluid Management With Mortality of Sepsis Patients With Congestive Heart Failure: A Retrospective Cohort Study
Source: Front Med (Lausanne). 2022 Mar 2;9:714384. doi: 10.3389/fmed.2022.714384 (PMC8924446; doi:10.3389/fmed.2022.714384)
Supplement: Supplementary file 1 [file Data_Sheet_1.docx]

Supplementary Material

**1 Supplementary Tables**

**1.1 Supplementary Table 1**

The International Classification of Diseases (ICD)-9 codes used for identifying patients with congestive heart failure and who underwent cardiovascular surgery with cardiopulmonary bypass

| **ICD-9 Codes** | **Disease reference** |
| --- | --- |
| **Heart failure** | |
| 398.91 | Rheumatic heart failure (congestive), rheumatic left ventricular failure |
| 402.01 | Malignant, with heart failure |
| 402.11 | Benign, with heart failure |
| 402.91 | Unspecified, with heart failure |
| 404.01 | Hypertensive heart and chronic kidney disease, malignant |
| 404.03 | Hypertensive heart and chronic kidney disease, malignant |
| 404.11 | Hypertensive heart and chronic kidney disease, benign |
| 404.13 | Hypertensive heart and chronic kidney disease, benign |
| 404.91 | Hypertensive heart and chronic kidney disease, unspecified |
| 404.93 | Hypertensive heart and chronic kidney disease, unspecified |
| 425.4 | Cardiomyopathy, other primary cardiomyopathies |
| 425.5 | Cardiomyopathy, alcoholic cardiomyopathy |
| 425.7 | Cardiomyopathy, nutritional and metabolic cardiomyopathy |
| 425.8 | Cardiomyopathy, cardiomyopathy in other disease classified elsewhere |
| 425.9 | Cardiomyopathy, secondary cardiomyopathy, unspecified |
| 428 | Heart failure |
| **Cardiopulmonary bypass** | |
| 39.61 | Cardiopulmonary bypass |

**1.2 Supplementary Table 2**

Percentage of missing values in variables

| Variables | Percentage (%) |
| --- | --- |
| Weight | 3.3 |
| White blood cells | 0.4 |
| Hemoglobin | 0.6 |
| Platelet | 0.4 |
| Ccr | 0.1 |
| BUN | 0.1 |
| Sodium | 0.1 |
| Potassium | 0.1 |
| Chloride | 0.1 |
| INR | 7.2 |
| Lactic acid | 21.9 |
| Heart rate | 0.1 |
| Mean MAP | 0.1 |
| Mean respiratory | 0.1 |
| Max temperature | 0.9 |
| SpO_2_ | 0.1 |

BUN, blood urea nitrogen; Ccr, creatinine clearance rate; INR, international normalized ratio; MAP, mean arterial pressure; SpO_2_, oxygen saturation

| **1.3 Supplementary Table 3**  AIC values of confounding variables explored with restricted cubic spline | | | |
| --- | --- | --- | --- |
|  | **Knots=3** | **Knots=4** | **Knots=5** |
| **Age** |  |  |  |
| AIC | 6.7 | 6.16 | 4.33 |
| Association p-value (Wald) | 0.006 | 0.008 | 0.017 |
| Linearity p-value (Wald) | 0.311 | 0.289 | 0.447 |
| **Weight** |  |  |  |
| AIC | 0.62 | 0.11 | 0.99 |
| Association p-value (Wald) | 0.111 | 0.112 | 0.141 |
| Linearity p-value (Wald) | 0.759 | 0.456 | 0.49 |
| **White blood cells** |  |  |  |
| AIC | -2.58 | 14.46 | 14.79 |
| Association p-value (Wald) | 0.485 | <0.001 | <0.001 |
| Linearity p-value (Wald) | 0.327 | <0.001 | <0.001 |
| **Hemoglobin** |  |  |  |
| AIC | 0.64 | -1.16 | -0.85 |
| Association p-value (Wald) | 0.103 | 0.191 | 0.135 |
| Linearity p-value (Wald) | 0.74 | 0.857 | 0.463 |
| **Platelets** |  |  |  |
| AIC | 18.39 | 16.8 | 15.09 |
| Association p-value (Wald) | <0.001 | <0.001 | <0.001 |
| Linearity p-value (Wald) | <0.001 | <0.001 | 0.003 |
| **Ccr** |  |  |  |
| AIC | 18.32 | 16.87 | 18.5 |
| Association p-value (Wald) | <0.001 | <0.001 | <0.001 |
| Linearity p-value (Wald) | 0.487 | 0.587 | 0.201 |
| **BUN** |  |  |  |
| AIC | 36.11 | 34.16 | 32.35 |
| Association p-value (Wald) | <0.001 | <0.001 | <0.001 |
| Linearity p-value (Wald) | 0.073 | 0.2 | 0.331 |
| **Na** |  |  |  |
| AIC | -0.48 | -1.37 | -2.79 |
| Association p-value (Wald) | 0.165 | 0.198 | 0.262 |
| Linearity p-value (Wald) | 0.286 | 0.316 | 0.414 |
| **K** |  |  |  |
| AIC | 3.54 | 2.64 | 1.86 |
| Association p-value (Wald) | 0.024 | 0.037 | 0.043 |
| Linearity p-value (Wald) | 0.951 | 0.58 | 0.508 |
| **INR** |  |  |  |
| AIC | 2.27 | 0.69 | - |
| Association p-value (Wald) | 0.044 | 0.081 | - |
| Linearity p-value (Wald) | 0.031 | 0.08 | - |
| **Heart rates** |  |  |  |
| AIC | -0.61 | -2.57 | -4.55 |
| Association p-value (Wald) | 0.18 | 0.326 | 0.481 |
| Linearity p-value (Wald) | 0.734 | 0.925 | 0.982 |
| **MAP** |  |  |  |
| AIC | 0.29 | 8.46 | 6.59 |
| Association p-value (Wald) | 0.118 | 0.002 | 0.005 |
| Linearity p-value (Wald) | 0.776 | 0.006 | 0.016 |

AIC, Akaike information criterion; BUN, blood urea nitrogen; Ccr, creatinine clearance rate; MAP, mean arterial pressure; Na, sodium; K, potassium; INR, international normalized ratio

**1.4 Supplementary Table 4**

Univariable logistic regression of confounding variables

| **Variables** | **Odds ratio** | **95% CI** | **p-value** |
| --- | --- | --- | --- |
| Age, years | 1.011 | 1.004–1.018 | 0.002 |
| Male | 1.081 | 0.88–1.33 | 0.457 |
| Weight (kg) | 0.995 | 0.991–1 | 0.036 |
| ICU types, n (%) |  |  |  |
| CCU as ref |  |  |  |
| CSRU | 0.93 | 0.535–1.571 | 0.791 |
| MICU | 0.972 | 0.752–1.261 | 0.831 |
| SICU | 0.768 | 0.523–1.118 | 0.173 |
| TSICU | 0.841 | 0.539–1.291 | 0.436 |
| SOFA first day | 1.148 | 1.107–1.19- | <0.001 |
| SAPS II first day | 1.043 | 1.034–1.052 | <0.001 |
| Comorbidities, n (%) |  |  |  |
| Hypertension | 0.794 | 0.646–0.977 | 0.029 |
| Diabetes with complication | 0.868 | 0.582–1.267 | 0.474 |
| Valvular disease | 0.905 | 0.69–1.18 | 0.468 |
| COPD | 1.053 | 0.847–1.306 | 0.64 |
| Kidney disease | 0.908 | 0.712–1.152 | 0.43 |
| Liver disease | 1.842 | 1.352–2.497 | <0.001 |
| Laboratory indexes |  |  |  |
| White blood cells (10^9^/L), max | 1.004 | 0.993–1.014 | 0.492 |
| Hemoglobin, min | 0.942 | 0.892–0.995 | 0.034 |
| Platelets, using RCS function | 0.998 | 0.998–0.999 | 0.003 |
| 57–143 | 0.994 | 0.998–1 | 0.033 |
| 43–194 | 1.006 | 0.961–1.054 | 0.786 |
| 194–256 | 1.022 | 0.828–1.262 | 0.839 |
| 256–405 | 0.94 | 0.723–1.22 | 0.642 |
| Platelets, using LSP function |  |  |  |
| ≤200 | 0.995 | 0.993–0.997 | <0.001 |
| >200 | 1.001 | 0.999–1.002 | 0.432 |
| Ccr | 0.994 | 0.991–0.997 | <0.001 |
| BUN, max | 1.012 | 1.008–1.016 | <0.001 |
| Na, min | 0.985 | 0.966–1.004 | 0.121 |
| K, min | 1.281 | 1.074–1.531 | 0.003 |
| INR, max | 1.039 | 0.977–1.103 | 0.205 |
| Lactic acid, max | 1.101 | 1.049–1.156 | <0.001 |
| Vital sign first day |  |  |  |
| Mean heart rates | 1.006 | 1–1.012 | 0.07 |
| Mean MAP | 0.992 | 0.984–1 | 0.04 |
| Dopamine, n (%) | 1.687 | 1.268–2.236 | <0.001 |
| Dobutamine, n (%) | 1.85 | 1.148–2.947 | 0.01 |
| Norepinephrine, n (%) | 1.745 | 1.396–2.179 | <0.001 |
| Epinephrine, n (%) | 0.556 | 0.128–1.714 | 0.359 |
| Vasopressor, n (%) | 1.534 | 1.247–1.889 | <0.001 |

CI, confidence interval; ICU, intensive care unit; CSRU, cardiac surgery intensive care unit; MICU, medicine intensive care unit; SICU, surgery intensive care unit; TSICU, trauma surgery intensive care unit; BUN, blood urea nitrogen; SOFA, Sequential Organ Failure Assessment; RCS, restricted cubic spline; Ccr, creatinine clearance rate; MAP, mean arterial pressure; LSP, linear spline

| **Variables** | **OR** | **95% CI** | **p-value** | **VIF** |
| --- | --- | --- | --- | --- |
| Age | 1.01 | 1.002−1.018 | 0.02 | 1.24 |
|  |  |  |  |  |
|  |  |  |  |  |
| SAPS II score | 1.029 | 1.018−1.04 | <0.001 | 1.442 |
| Hypertension | 0.754 | 0.603−0.943 | 0.013 | 1.047 |
| Liver disease | 1.629 | 1.15−2.3 | 0.006 | 1.134 |
| Platelets (per 10^9^/L) |  |  |  | 1.124 |
| ≤200 | 0.996 | 0.993−0.998 | <0.001 |  |
| >200 | 1 | 0.999−1.002 | 0.32 |  |
| BUN | 1.007 | 1.002−1.011 | 0.003 | 1.103 |
| MAP | 1.008 | 0.999−1.018 | 0.077 | 1.17 |
| Fluid accumulation index (per 48 hours) | |  |  | 1.195 |
| ≤0 | 1.408 | 1.088−1.915 | 0.017 |  |
| >0 and ≤0.42 | 0.584 | 0.23−1.481 | 0.258 |  |
| >0.42 | 4.683 | 2.018−10.927 | <0.001 |  |
| Dopamine | 1.363 | 0.999−1.85 | 0.048 | 1.056 |

**1.5 Supplementary Table 5**

Multivariable logistic variable regression using linear spline function, AIC =1978

CI, confidence interval; OR, odds ratio; BUN, blood urea nitrogen; MAP, mean arterial pressure; SAPS II, Simplified Acute Physiology Score II; AIC, Akaike information criterion; VIF, Variance inflation factor

**1.6 Supplementary Table 6**

Multivariable logistic variable regression using designed variables, AIC=1988

| **Variables** | **OR** | **95% CI** | **p-value** | **VIF** |
| --- | --- | --- | --- | --- |
| Age | 1.008 | 1.0−1.016 | 0.045 | 1.109 |
| SAPS II | 1.032 | 1.022−1.042 | <0.001 | 1.153 |
| Hypertension | 0.747 | 0.598−0.931 | 0.01 | 1.022 |
| Liver disease | 1.649 | 1.167−2.321 | 0.004 | 1.066 |
| Platelet counts (per 10^9^/L) |  |  |  | 1.028 |
| ≤200 | 0.996 | 0.994−0.998 | 0.001 |  |
| >200 | 1.001 | 0.999−1.002 | 0.28 |  |
| BUN | 1.007 | 1.003−1.011 | 0.002 | 1.05 |
| Fluid accumulation index |  |  |  | 1.028 |
| <0 | 1.084 | 0.779−1.509 | 0.634 |  |
| 0–0.42 as reference | - | - | - |  |
| >0.42 | 1.472 | 1.111−1.963 | 0.008 |  |

CI, confidence interval; OR, odds ratio; BUN, blood urea nitrogen; MAP, mean arterial pressure; SAPS II, Simplified Acute Physiology Score II; AIC, Akaike information criterion; VIF, Variance inflation factor

**1.7 Supplementary Table 7**

Multivariable logistic regression when FB was forced into the regression model

| **Variables** | **OR** | **95% CI** | **p-value** |
| --- | --- | --- | --- |
| FB (per mL/kg/48 h) |  |  |  |
| ≤0 | 0.875 | 0.658−1.161 | 0.358 |
| 0−60 as reference |  |  |  |
| >60 | 1.168 | 0.901−1.513 | 0.239 |
| Age | 1.01 | 1.002−1.018 | 0.017 |
| SAPS II | 1.032 | 1.021−1.043 | <0.001 |
| Hypertension | 0.754 | 0.604−0.941 | 0.013 |
| Platelet counts (per 10^9^/L) |  |  |  |
| ≤200 | 0.996 | 0.994−0.998 | <0.001 |
| >200 | 1.001 | 0.999−1.002 | 0.3 |
| BUN | 1.007 | 1.003−1.011 | 0.002 |
| Sodium | 0.986 | 0.966−1.005 | 0.156 |
| MAP | 1.009 | 1.000−1.019 | 0.05 |
| Dopamine | 1.296 | 0.948−1.761 | 0.1 |
| Norepinephrine | 1.267 | 0.981−1.634 | 0.068 |

CI, confidence interval; OR, odds ratio; BUN, blood urea nitrogen; MAP, mean arterial pressure; SAP SII, Simplified Acute Physiology Score II; FB, fluid balance

**1.8 Supplementary Table 8**

Multivariable logistic regression when FI was forced into the regression model

| **Variables** | **OR** | **95% CI** | **p-value** |
| --- | --- | --- | --- |
| FI (per mL/kg/48 h) |  |  |  |
| ≤45 | 0.815 | 0.615−1.075 | 0.15 |
| 45−100 as reference |  |  |  |
| >100 | 1.064 | 0.819−1.378 | 0.641 |
| Age | 1.009 | 1.000−1.017 | 0.029 |
| SAPS II | 1.033 | 1.022−1.044 | <0.001 |
| Hypertension | 0.759 | 0.608−0.948 | 0.015 |
| Liver disease | 1.626 | 1.149−2.291 | 0.006 |
| Platelet counts (per 10^9^/L) |  |  |  |
| ≤200 | 0.996 | 0.994−0.998 | <0.001 |
| >200 | 1.000 | 0.999−1.002 | 0.277 |
| BUN | 1.007 | 1.003−1.011 | 0.002 |
| MAP | 1.009 | 1.000−1.018 | 0.065 |
| Dopamine | 1.322 | 0.970−1.795 | 0.075 |
| Norepinephrine | 1.266 | 0.980−1.632 | 0.07 |

CI, confidence interval; OR, odds ratio; BUN, blood urea nitrogen; MAP, mean arterial pressure; SAPS II, Simplified Acute Physiology Score II; FI, fluid intake

**Supplementary Figure legends**

Supplementary Figure 1. Flow chart of the present study.

ICU, intensive care unit; CHF, congestive heart failure; RRT, renal replacement therapy

Supplementary Figure 2. Relationships among fluid intake, fluid balance, fluid accumulation index and in-hospital mortality explored by restricted cubic spline function.

AIC, Akaike information criterion

Supplementary Figure 3. Relationships between other confounding factors and in-hospital mortality explored by restricted cubic spline function.

CCR, creatinine clearance rate; MAP, mean arterial pressure
