# Supplementary figures and images for: Association of Fluid Management With Mortality of Sepsis Patients With Congestive Heart Failure: A Retrospective Cohort Study
Source: Front Med (Lausanne). 2022 Mar 2;9:714384. doi: 10.3389/fmed.2022.714384 (PMC8924446; doi:10.3389/fmed.2022.714384)

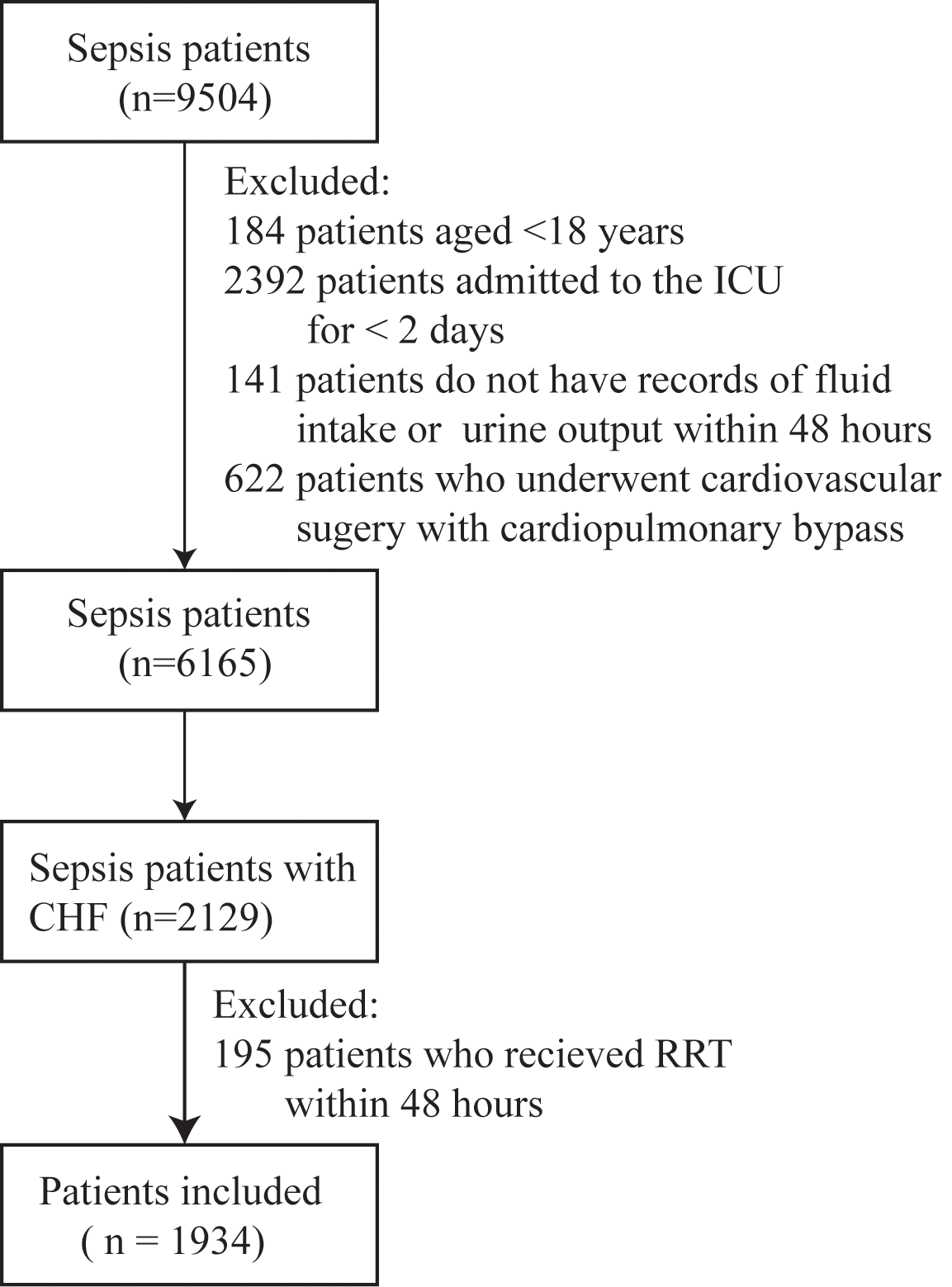

Supplement: Supplementary file 2 [file Image_1.TIF]

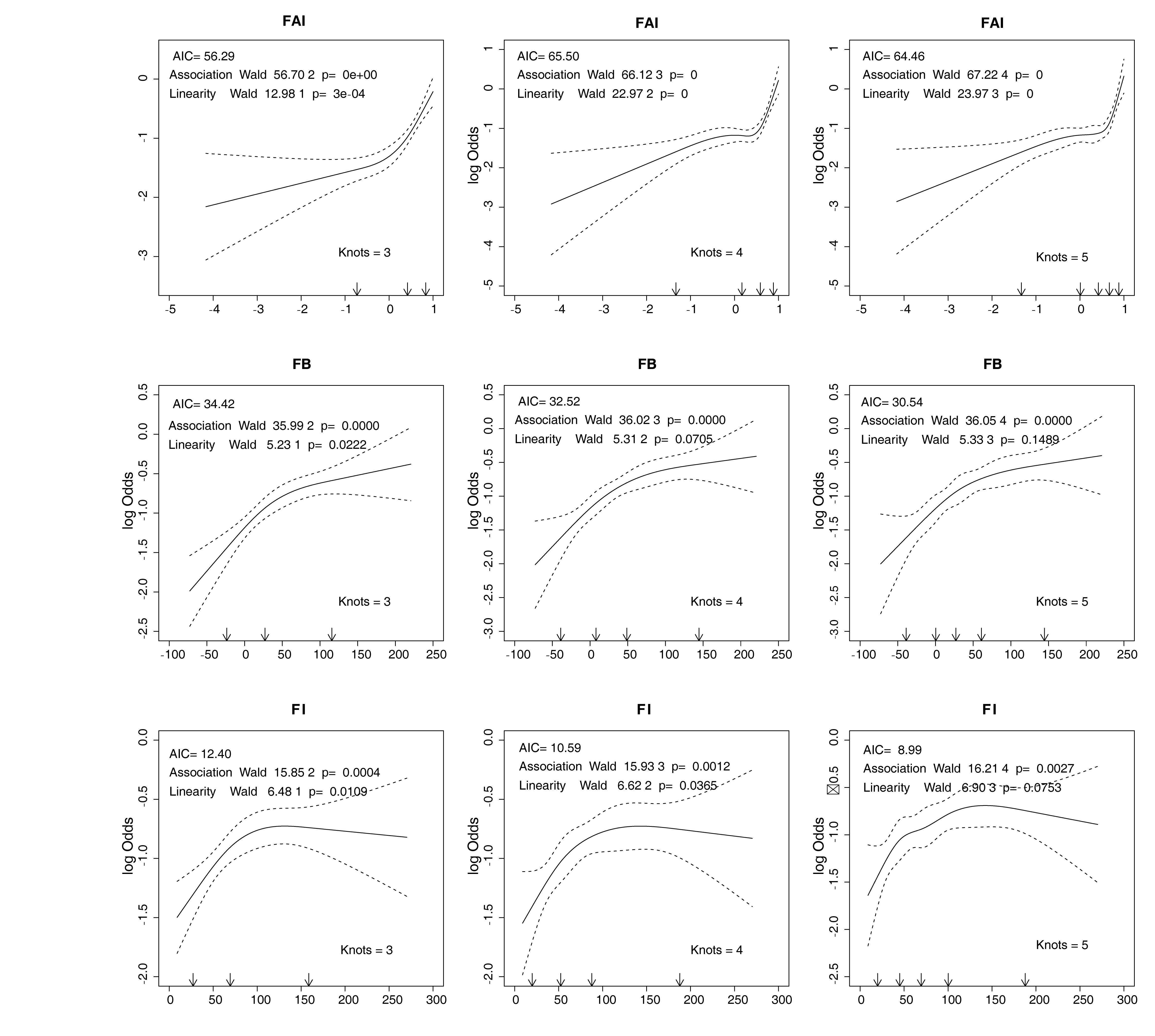

Supplement: Supplementary file 3 [file Image_2.JPEG]

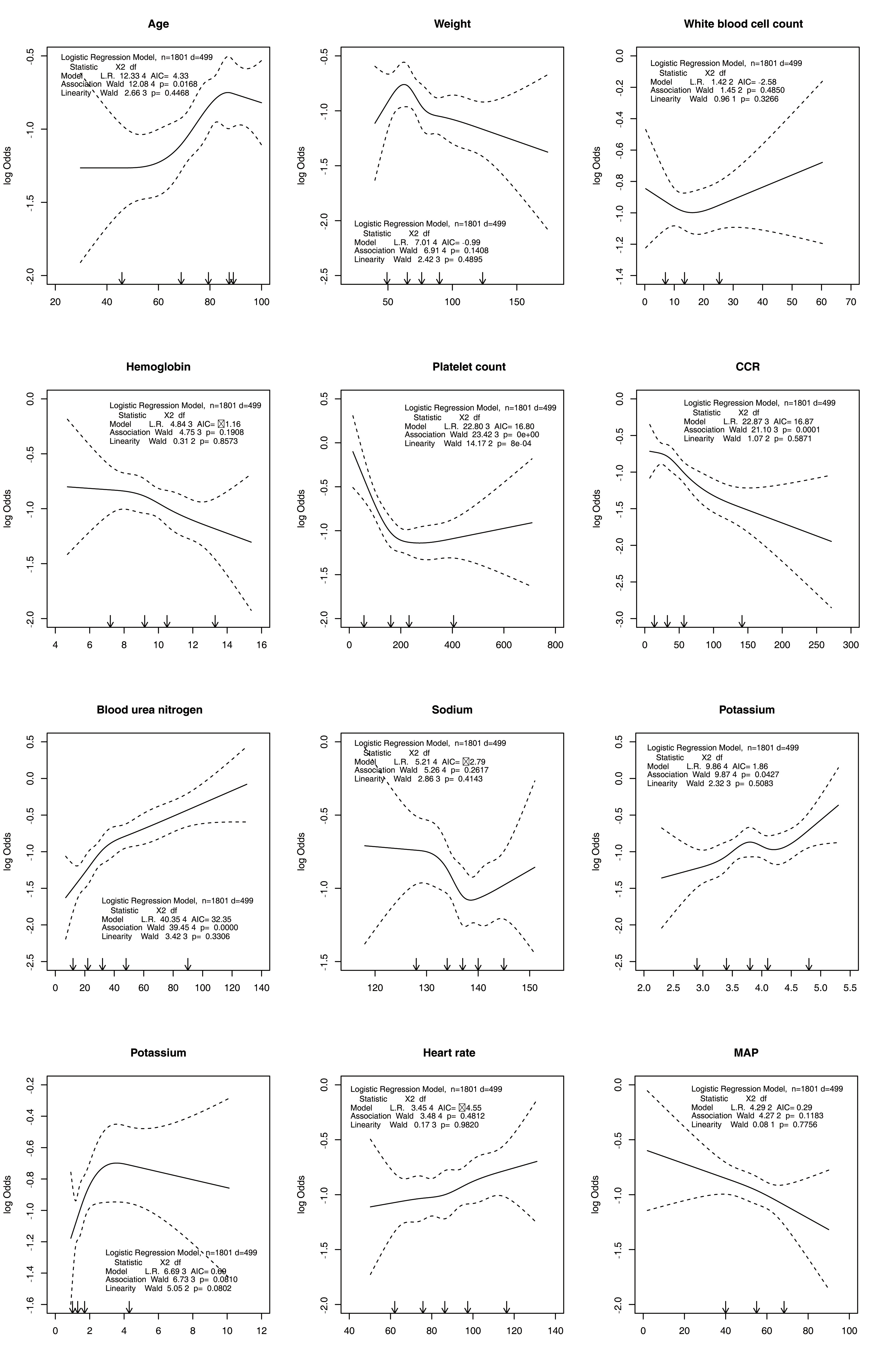

Supplement: Supplementary file 4 [file Image_3.JPEG]
